# The Role of Surgical Approaches in the Multi-Modal Management of Adult Craniopharyngiomas

**DOI:** 10.3390/curroncol29030118

**Published:** 2022-02-24

**Authors:** Christopher S. Hong, Sacit Bulent Omay

**Affiliations:** Department of Neurosurgery, Yale School of Medicine, New Haven, CT 06510, USA; christopher.hong@yale.edu

**Keywords:** craniopharyngioma, endoscopic endonasal approach, transphenoidal, supraorbital, ommaya, radiation

## Abstract

Craniopharyngiomas are rare, benign primary brain tumors that arise from remnants of the craniopharyngeal duct epithelium within the sellar and suprasellar region. Despite their benign biology, they may cause significant morbidity, secondary to involvement of nearby eloquent neural structures, such as the pituitary gland, hypothalamus, and optic apparatus. Historically, aggressive surgical resection was the treatment goal to minimize risk of tumor recurrence via open transcranial midline, anterolateral, and lateral approaches, but could lead to clinical sequela of visual, endocrine, and hypothalamic dysfunction. However, recent advances in the endoscopic endonasal approach over the last decade have mostly supplanted transcranial surgery as the optimal surgical approach for these tumors. With viable options for adjuvant radiation therapy, targeted medical treatment, and alternative minimally invasive surgical approaches, the management paradigm for craniopharyngiomas has shifted from aggressive open resection to more minimally invasive but maximally safe resection, emphasizing quality of life issues, particularly in regards to visual, endocrine, and hypothalamic function. This review provides an update on current multi-modal approaches for craniopharyngiomas, highlighting the modern surgical treatment paradigm for this disease entity.

## 1. Introduction

Craniopharyngiomas are uncommon, benign central nervous system (CNS) tumors that arise from the remnants of the craniopharyngeal duct epithelium in the sellar and suprasellar region [1]. They represent approximately 2–5% of all primary intracranial tumors [2,3]. Despite their benign biological behavior, they may cause significant morbidity, secondary to the proximity of eloquent neural structures, such as the pituitary gland, hypothalamus, and optic apparatus. While adjuvant chemotherapy and/or radiation has limited efficacy, maximal surgical resection remains the mainstay of therapy. Current surgical treatment paradigms have evolved to tailor endoscopic endonasal, transcranial, or combined endoscopic endonasal and transcranial approaches to maximally resect craniopharyngiomas, based on individual tumor characteristics. This review discusses modern surgical approaches and adjunctive therapeutic modalities for the treatment of craniopharyngioma.

### 1.1. Epidemiology and Clinical Presentation

The incidence of craniopharyngiomas is between 1.3 to 1.7 cases per 1,000,000 person-years with a bimodal age distribution [2,3]. Approximately, 1.9 cases per 1,000,000 person-years occur in children ages 0–19 with a second peak of 2.1 cases per 1,000,000 person-years in adults aged 40–79 [4]. Patients present with symptoms related to compression of nearby eloquent structures, including visual deficits from compression of the optic nerves, hormonal imbalances from pituitary gland/stalk invasion, cognitive changes from frontal lobe involvement and, particularly in children, behavioral or developmental delay secondary to hypothalamic spread [4,5]. Headaches are also a common complaint, due to increased intracranial pressure secondary to obstructive hydrocephalus [4].

### 1.2. Radiologic and Histopathologic Features

There are two histological subtypes of craniopharyngioma: adamantinomatous and papillary. While adamantinomatous craniopharyngiomas can occur in children and adults, the papillary subtype is mainly observed in adults [1,6]. The adamantinomatous subtype is thought to arise from neoplastic transformation of epithelial cell remnants within the craniopharyngeal duct. Histologically, they exhibit palisading a columnar epithelium surrounding well-differentiated squamous epithelium, wet keratin, and stellate reticulum associated with adjacent gliosis and Rosenthal fibers [7]. Activating mutations of the WNT pathway gene, CTNNB1, characterize the majority of cases [8] while rare downstream WNT pathway mutations in APC have also been described [9]. Papillary craniopharyngiomas are thought to arise from transformed epithelial cells within the pituitary stalk. Histologically, they exhibit papillary architecture, characterized by solid sheets of well-differentiated non-keratinizing squamous epithelium and fibrovascular cores surrounded by crude papillae [7]. Nearly all cases of papillary craniopharyngioma demonstrate BRAF V600E mutations [10], which may be amenable to treatment with anti-BRAF agents and can lead to dramatic tumor response [11].

On imaging, most craniopharyngiomas measure between 2–4 cm at time of diagnosis and demonstrate a cystic component in approximately half of cases, more prevalent in the adamantinomatous subtype [12,13]. On magnetic resonance imaging (MRI), the solid component of craniopharyngiomas is variable and can range from hypointense to hyperintense on T1-weighted and T2-weighted imaging [14], depending on the presence or absence of calcifications. The presence of calcifications can be better evaluated with computed tomography (CT) and correlates more commonly with the adamantinomatous variant [1]. After administration of contrast, there is frequently enhancement of the cyst wall as well as heterogeneous enhancement of the solid portion of the tumor.

## 2. Surgical Principles and Approaches

Despite being a benign tumor, craniopharyngiomas can cause significant morbidity, related to visual, endocrine, hypothalamic, and cognitive dysfunction. As such, the primary goal of surgery is to decompress involved structures, while achieving maximally safe resection. If possible, gross total resection (GTR) is the best surgical outcome, as recurrence rates are significantly lower in cases of GTR [15]. Recent large case series have reported rates of GTR ranging between 37.5–91% with recurrence rates between 2–35% [16,17,18,19,20,21,22,23,24]. Likewise, a recent meta-analysis revealed GTR rates of 67.8% for endoscopic endonasal resections of craniopharyngiomas with recurrence rates of 21% [25]. However, GTR often requires sacrifice of the pituitary stalk, leading to anterior pituitary dysfunction and diabetes insipidus, the latter occurring in 50% of patients even when the pituitary stalk is preserved [21]. In addition, GTR is often precluded by a tumor adherent to neurovascular structures, the optic nerves, and hypothalamus, in which inadvertent injury can lead to permanent morbidity from stroke, visual deficits, and hypothalamic dysfunction. In particular, hypothalamic obesity from iatrogenic injury can be devastating for patients with significant metabolic sequela and unfortunately is not particularly responsive to pharmacologic therapies or bariatric treatments [1,26]. As such, the modern surgical approach to craniopharyngiomas has been focused on maximally safe resection while minimizing postoperative complications, particularly in regard to sparing the optic nerves and hypothalamus. Compared to GTR, subtotal resection (STR) followed by adjuvant radiation has demonstrated similar rates of local control, and has even been suggested by some groups to be the preferred treatment strategy [27,28,29,30].

Historically, craniopharyngiomas were resected via open transcranial techniques, later improved by the introduction of the surgical microscope [31,32]. The most common transcranial approaches are unilateral pterional, frontolateral/supraorbital, and orbitozygomatic approaches, as well as midline subfrontal or transcallosal approaches [33]. While open approaches allow for greater freedom of movement, brain retraction is required in and around the optic nerve and nearby vascular structures, and importantly, only provides limited exposure below the chiasm and behind the sella into the interpeduncular cisterns [5,34]. Over the last two decades, the development of the endoscopic endonasal approach has revolutionized anterior skull base surgery, providing improved resolution and maneuverability compared to microscopic open and transnasal surgery. Initially, endoscopic endonasal approaches were limited to pituitary adenomas and craniopharyngiomas within the sellar region. However, with improved understanding of anterior skull base anatomy and endoscopic technologies, endoscopic endonasal approaches have evolved to afford excellent access to the suprasellar region and even extending into the third ventricle [35,36]. Multiple systematic reviews have suggested an improved GTR rate with the endoscopic endonasal approach compared to open transcranial surgery [25,37] with rates of GTR after the endoscopic endonasal approach ranging anywhere from 37.5% to 91% [16,17,18,19,20,21,22,23,24]. Likewise, they found improved visual outcomes and lower complication rates related to panhypopituitarism, diabetes insipidus, and seizures with the endoscopic endonasal approach [37,38]. While there were higher rates of cerebrospinal fluid leakage with the endoscopic endonasal approach, more recent studies have demonstrated significantly lower rates of cerebrospinal fluid leak, attributed to improved skull base reconstruction techniques and, most importantly, use of the vascularized nasoseptal flap [34,39]. Key advantages and disadvantages of the endoscopic endonasal approach are outlined in Table 1.

The supraorbital keyhole craniotomy is an alternative minimally invasive surgical approach to craniopharyngiomas in the suprasellar region and around the third ventricle. This approach may afford wider visualization of the internal carotid arteries, optic chiasm and nerves, optico-carotid recess, lamina terminalis, and pituitary stalk, while minimizing the risk of cerebrospinal fluid leak, associated with an expanded endoscopic endonasal approach [40]. In general, tumors with lateral extension beyond the optic canal or supraclinoid internal carotid arteries may be more amenable to a supraorbital approach that affords better visualization of the lateral aspects of the tumor [41]. Others have described combining the endoscopic endonasal approach and supraorbital craniotomy in a single setting for patients with parasellar lesions extending laterally beyond the internal carotid artery and/or are fibrous and firmly adhered to nearby eloquent neurovascular structures [42]. This approach may yield higher rates of decompression of compressed visual structures, while minimizing surgical complications related to pituitary dysfunction, diabetes insipidus, and/or cerebrospinal fluid leakage [42,43].

## 3. Description of Surgical Techniques

### 3.1. Endoscopic Endonasal Approach

Here, we provide a succinct overview of our surgical technique for the endoscopic endonasal approach for craniopharyngiomas. With the ability to expand the endoscopic endonasal approach beyond the transphenoidal approach into the transtuberculum and transplanum region, we have found that the majority of craniopharyngiomas are amenable to the endoscopic endonasal approach alone. In cases where there is significant lateral expansion of the tumor, limiting endoscopic visualization, we consider adjunct surgical approaches after initial endoscopic endonasal debulking.

**Figure 1:**
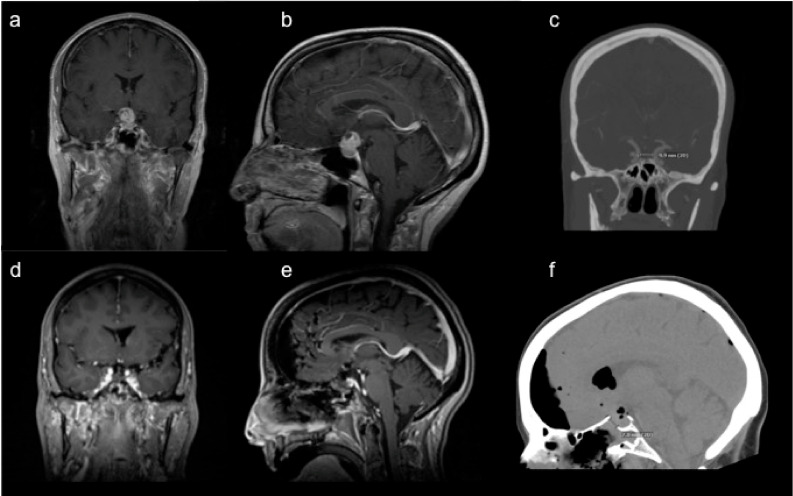
Relevant peri-operative imaging. A 67-year-old female presented with subjective decline in vision over several months and was diagnosed with bitemporal hemianopsia. Representative (**a**) coronal and (**b**) sagittal T1-weighted post-contrast magnetic resonance image (MRI) revealed a heterogeneously enhancing 1.7 cm sellar/suprasellar mass, consistent with a diagnosis of craniopharyngioma. (**c**) Pre-operative coronal computed tomography angiography (CTA) demonstrated an approximate 1 cm inter-carotid distance, relevant for surgical planning. Representative post-operative (**d**) coronal and (**e**) sagittal T1-weighted post-contrast MRI showed gross total resection through a (**f**) minimally invasive sub-centimeter craniotomy via an endoscopic endonasal transphenoidal approach, as shown on sagittal CT.

All patients undergo standard pre-operative evaluation to assess visual and endocrine function by our neuro-opthalmology and endocrine colleagues. This includes formal visual field testing and measurement of pituitary hormone levels. A dedicated pituitary protocol MRI and CT angiography (CTA) of the head are obtained to understand the locations of the pituitary gland/stalk, optic nerves, internal carotid arteries, and anterior cerebral and middle cerebral arteries in relationship to the tumor (Figure 1a–c).

The technique of the endoscopic endonasal approach has been extensively described in the literature [44,45,46]. Here, we highlight key surgical steps and nuances, practiced at our institution. Like most multi-disciplinary centers, our otolaryngology colleagues begin the endonasal approach to gain adequate exposure of the anterior skull base. We routinely use neuro-navigation as an adjunct to gross identification of important anatomic landmarks, including the tuberculum sella anteriorly, the clival recess posteriorly, and the cavernous internal carotid arteries and lateral optico-carotid recesses laterally. The location of the internal carotid arteries is further confirmed with the use of a vascular Doppler.

**Figure 2:**
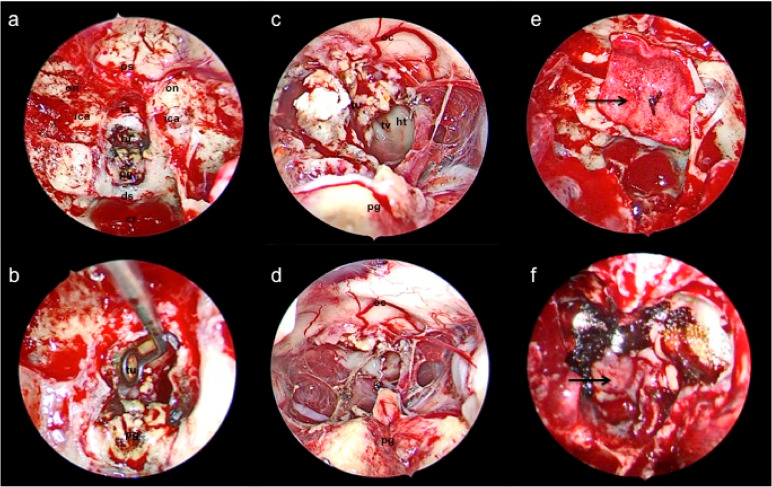
Intra-operative images from the endoscopic endonasal approach. (**a**) An expanded view after craniotomy and dural opening reveals relevant anatomy, followed by (**b**) a close-up view illustrating use of an angled curette for tumor resection. The anatomy of nearby critical neural structures is shown after (**c**) tumor debulking, followed by (**d**) gross total resection. (**e**) The button double-layer closure technique with an acellular dermal matrix (arrow) is shown (**f**) reinforced with nasoseptal flap (arrow) coverage that is postage-stamped with absorbable hemostatic agents. Abbreviations: cr (clival recess), ds (dorsum sphenoidale), ht (hypothalamus), ica (internal carotid artery), oc (optic chiasm), on (optic nerve), pg (pituitary gland), ps (planum sphenoidale), ts (tuberculum sphenoidale), tu (tumor), tv (third ventricle).

Subsequently, a bimanual approach is performed through each nasal cavity during the neurosurgical portion of the operation, aided by endoscopic visualization by otolaryngology. Afer routine craniotomy and dural opening, the corridor between the optic chiasm and pituitary gland is dissected to approach the tumor capsule. (Figure 2a). The tumor capsule is then coagulated and opened with endoscopic scissors and subsequently resected in piecemeal fashion with the use of cup forceps, suction, and an ultrasonic surgical aspirator (Figure 2b). A series of 0, 30, and 45 degree-angled endoscopes are used to visualize the surgical cavity, as guided by neuronavigation. Care is taken to preserve branches of the superior hypophyseal artery complex, as well as the pituitary stalk, and floor of the third ventricle formed by the hypothalamus (Figure 2c). A residual tumor is intentionally left behind if it is adherent to eloquent structures, such as the optic nerves or hypothalamus.

After the tumor is maximally resected (Figure 2d), we typically perform an intra-operative MRI to confirm decompression of the optic nerves and assess for any residual tumor that can be safely resected (Figure 1d,e). The latter is rarely the case, as the angled endoscopes facilitate adequate visualization of the surgical cavity. Subsequently, attention is turned towards reconstruction of the anterior skull base defect. The skull base is reconstructed with a button double-layer closure [47] with a dural substitute covering it as an inlay and an overlay (Figure 2e), followed by coverage over the construct with the harvested nasoseptal flap (Figure 2f). A head CT is routinely obtained as a baseline post-operative image (Figure 1f). 

### 3.2. Supraorbital Craniotomy

We have utilized the supraorbital approach as an ideal surgical corridor for the majority of craniopharyngiomas with significant antero-lateral extension in the suprasellar region beyond the optic chiasm and supraclinoid internal carotid arteries, respectively. In contrast, more midline and sellar-based craniopharyngiomas can be readily removed with the endoscopic endonasal approach alone. Given the versatility of an expanded endoscopic endonasal approach, we resect the vast majority of craniopharyngiomas endoscopically as the initial approach and reserve the supraorbital craniotomy as a surgical adjunct for those with significant laterally located residual tumors, causing persistent compression of eloquent structures. A brief description of our surgical technique is described below.

**Figure 3:**
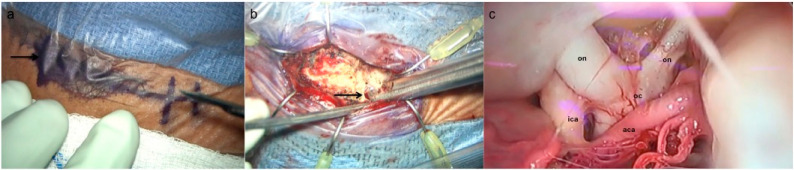
Intra-operative images from a supraorbital craniotomy. (**a**) The planned incision is illustrated, beginning just lateral to the supraorbital notch (arrow) within the eyebrow and extending laterally. (**b**) After inferior reflection of the orbicularis and temporalis muscle flap, a burr hole is made at the keyhole (arrow) and further widened with a rongeur. (**c**) A endoscopic cadaver view demonstration of the supraorbital approach to the suprasellar area. The internal carotid artery, optic nerves, optic chiasm, and the anterior cerebral arteries are shown. Abbreviations: aca (anterior cerebral artery), ica (internal carotid artery), oc (optic chiasm), on (optic nerve).

The supraorbital approach has been described in detail previously in the literature [48,49,50]. Key surgical considerations include planning the eyebrow incision, lateral to the supraorbital notch to avoid injury to the supraorbital nerve (Figure 3a). The craniotomy is performed via a single burr hole at the keyhole (Figure 3b) and typically measures 2–2.5 cm in width and 1.5–2 cm in height. Further drilling of the supraorbital rim is performed to be flush with the floor of the anterior fossa to expand the surgical field. After standard dural opening, a subfrontal dissection is performed microscopically towards the suprasellar region. Opening of the chiasmatic and carotid cisterns to permit cerebrospinal fluid egress may further relax the frontal lobe and avoid need for retraction. If the subfrontal corridor remains limited despite this maneuver, a frontal ventriculostomy can be performed under neuronavigation to drain off cerebrospinal fluid. The tumor is subsequently resected in routine fashion, utilizing a combination of suction, curettes, bipolar electrocautery, and occasionally the use of an ultrasonic aspirator for particularly fibrous tumors. 

## 4. Adjunctive Treatment Modalities

### 4.1. Stereotactic Cyst Decompression

A subset of craniopharyngiomas cause symptoms related to mass effect from the cystic component of the tumor, either at time of diagnosis or tumor recurrence. In these cases, alternative strategies aimed at reducing the cyst via stereotactic or neuroendoscopic cyst aspiration may be a viable strategy with less morbidity than surgical resection [51,52]. Most commonly, this has been done with the Ommaya reservoir system, which enables repeated aspiration of the cyst fluid through a subcutaneous reservoir. The device also allows for injection of chemotherapies into the cyst and/or tumor through the same reservoir, typically with bleomycin or interferon alpha, with widely varying cyst control rates ranging from 14–78% [53,54,55]. 

In the pediatric population, the use of the Ommaya reservoir system has been described to treat mass effect-related symptoms and delay the need for aggressive surgical surgery and/or radiation to allow for full maturation of the hypothalamic-pituitary axis [56]. This application has been particularly studied in intra-cavitary brachytherapy, where a radioisotope is injected into the tumor cyst cavity, typically through an Ommaya reservoir. The most common radioisotopes are beta-emitting agents, including phosphorus-32 (^32^P), yttrium-90 (^90^Y), resulting in radiation dosages, ranging between 150–500 Gy, delivered to the cyst wall [57,58]. A recent systematic review of intra-cavitary brachytherapy in cystic craniopharyngiomasv [58] summarized findings from eight retrospective trials comprising 228 patients (66 children) and found tumor reduction responses in approximately 70% of cases, which was consistent with smaller historical trials [59,60,61,62,63]. However, treatment response was much more pronounced in purely cystic versus mixed solid-cystic lesions, particularly in the pediatric population (89% vs. 58%, respectively) [58]. Higher rates of visual (64% vs. 48%) and endocrine (20% vs. 7%) improvement were also observed in cystic versus mixed solid-cystic tumors but only when analyzing pediatric trials alone, as opposed to inclusion of mixed adult/pediatric trials. As such, while the literature remains limited, the Ommaya reservoir may serve as an important adjunctive treatment, particularly for pediatric patients with cystic craniopharyngiomas, for delivery of intra-cavitary brachytherapy to delay or obviate the need for more aggressive surgical resection and/or radiosurgery.

In the adult population, the use of Ommaya reservoirs for cystic craniopharyngiomas as an isolated therapy has been mainly limited to smaller and heterogeneous case series with relatively low recurrence rates of 0–27.3% over a follow-up period up to 56 months [51,64,65]. The largest series was reported by Moussa et al. who treated 52 patients (36 pediatric and 16 adults) with no prior resection, achieving local tumor control in 73% of cases at mean follow-up of 54 months [65]. Only 19% of patients required re-aspiration every 6 months, while the lack of need for re-aspiration in the remaining patients was attributed to marsupialization of the cyst after initial collapse into surrounding cerebrospinal fluid spaces via the Ommaya catheter holes. A more recent study by Steiert et al. validated this finding in 12 patients with cystic craniopharyngiomas who underwent stereotactic catheter placement into the ventricular system with the tip in the cyst, thus creating a cysto-ventricular shunt. This resulted in mean reduction of cyst volume of 92% at median follow-up of 41 months [66]. 

The Ommaya catheter can be inserted under neuroendoscopic visualization, stereotactically, or under direct visualization during open surgery. A recent case series of 11 adult patients by Frio et al. did not find any significant differences in local cyst growth control with either endoscopic or stereotactic reservoir insertion techniques [51]. Stereotaxy was employed in extraventricular cysts without co-existing hydrocephalus. In cotrast, endoscopy was useful for predominantly intraventricular and/or hydrocephalus-causing lesions, in which the cyst wall could be fenestrated and marsupialized into the ventricles or basal cisterns. Indeed, endoscopic cyst fenestration with placement of a Ommaya reservoir has been well-described in the management of pediatric cystic craniopharyngiomas with or without hydrocephalus as a means to avoid the risks of aggressive surgical resection during childhood development [67,68,69,70]. Although not as well described, this approach has been applied in the adult population, particularly for patients with tumors deemed to be highly unresectable or with medical co-morbidites, precluding aggressive surgical intervention. These studies include description of endoscopic cyst fenestration with Ommaya reservoir placement with or without radiotherapy as an effective first-line treatment strategy in these patients [65,71,72,73,74,75]. As such, this treatment strategy may be particularly relevant for adult patients with cystic craniopharyngiomas who present with concomitant hydrocephalus or who are not candidates for maximal surgical resection. 

**Figure 4:**
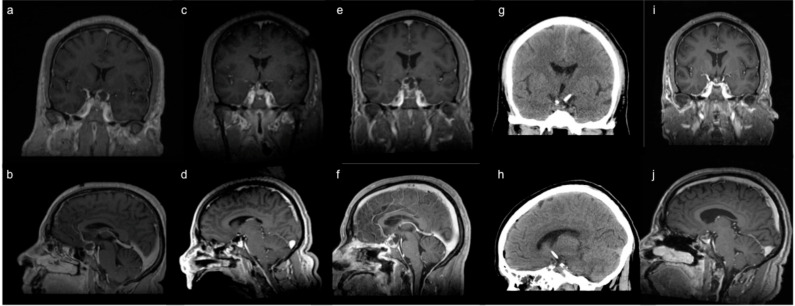
Ommaya reservoir placement for cystic recurrence. A 52-year-old male was diagnosed with a cystic suprasellar lesion on work-up for progressive left sided visual deficits, consistent with craniopharyngioma. Representative (**a**) coronal and (**b**) sagittal T1-weighted post-contrast MRI showed a predominantly cystic, enhancing 1.5 cm suprasellar lesion compressing the optic nerves, eccentric to the left. Intra-operative (**c**) coronal and (**d**) sagittal T1-weighted post-contrast MRI after resection demonstrated decompression of the optic chiasm and nerves with known adherent residual tumor left intentionally along the cavernous sinus. (**e**) Coronal and (**f**) sagittal T1-weighted post-contrast MRI obtained 5 months after initial resection showed cystic recurrence in the setting of recurrent visual decline. This was treated with Ommaya reservoir placement, as depicted on (**g**) coronal and (**h**) sagittal post-operative head CT, followed by adjuvant stereotactic radiosurgery. (**i**) Coronal and (**j**) sagittal T1-weighted post-contrast MRI obtained 3 months after cyst drainage and adjuvant radiation demonstrated durable cyst decompression with improvement of the patient’s visual symptoms.

The addition of adjuvant radiotherapy may facilitate local tumor control after Ommaya catheter placement for cystic craniopharyngiomas [73,74,75,76]. A recent case series of six patients by Laville et al. found favorable local tumor control at 12-month follow-up after combined Ommaya reservoir placement and hypofractionated radiotherapy at lower doses of radiation than would be required had the cyst not been drained [77]. Likewise, Jarebi et al. also described a patient in whom stereotactic Ommaya reservoir placement and adjuvant radiosurgery of the solid component of a cystic craniopharyngioma successfully alleviated visual deficits and intracranial hypertension without the need for open surgery [71]. At our institution, we typically reserve use of the Ommaya reservoir for recurrent cystic craniopharyngiomas after first-line surgical resection. In particular, we have found durable response rates with cyst drainage by the Ommaya reservoir followed by adjuvant stereotactic radiosurgery (Figure 4). Moreover, we have found that reduction of the cyst allows for higher, more focused radiation doses to the solid tumor, while minimizing radiation exposure to nearby eloquent structures such as the optic apparatus and brainstem. 

### 4.2. Radiation

Radiotherapy is an alternative treatment for craniopharyngiomas, either used alone or in combination with surgery. Traditionally, conventional external beam radiotherapy using X-rays was used but at the expense of collateral radiation exposure to normal tissues from the entering and exiting traversing beams [78,79]. Proton radiation therapy has allowed for more focal targeting of the tumor but has been limited by relatively few centers offering this treatment and may also lead to long-term cognitive, visual, and endocrine deficits particularly in children [80]. With advances in neuroimaging techniques, stereotactic radiosurgery has emerged as a favorable technique for delivering targeted radiation doses to the tumor while sparing nearby eloquent structures such as the optic pathway, hypothalamus, pituitary gland, and cerebral vasculature. Larger case series with long-term follow-up in patients undergoing stereotactic radiosurgery for previously operated tumors have demonstrated tumor control rates of between 60.8–76% at 5 years and 42.6–60.2% at 10 years [81,82,83,84,85,86,87]. However, radiation dosing must be carefully planned as adequate doses greater than 12 Gy have been shown to yield better local control [88] but maximum dosing exceeding 35 Gy may lead to tissue toxicity [85]. Adverse radiation rates in larger case series have ranged from 6.2–10.9%, most commonly hypopituitarism, followed by visual deficits and hypothalamic obesity [83,86]. Furthermore, radiation therapy may not address the cystic component of craniopharyngiomas, which may require surgical intervention in the form of stereotactic or neuroendoscopic drainage beforehand or at the time of radiation in cases of large cysts causing symptoms [89,90,91].

In general, radiotherapy in craniopharyngiomas has been utilized as an adjuvant therapy after STR, leading to similar tumor recurrence rates of 9.1–37.5%, compared to those following GTR alone [30,92,93,94]. This is particularly salient as an overly aggressive surgical approach to achieve GTR may lead to debilitating surgical morbidity, particularly involving the optic nerve and hypothalamic-pituitary axis [28,30]. After STR though, it remains to be seen whether radiation is best reserved for immediately after resection or at time of tumor progression. To date, studies have not demonstrated any significant differences in local tumor control or overall survival [95,96,97,98]. Likewise, the efficacy of radiotherapy as a single treatment modality upfront for craniopharyngiomas has not been well-characterized and only limited to case reports [99,100] and as such is typically reserved for patients who are otherwise medically unfit for surgery [101].

## 5. Overall Approach to Surgical Treatment

**Figure 5:**
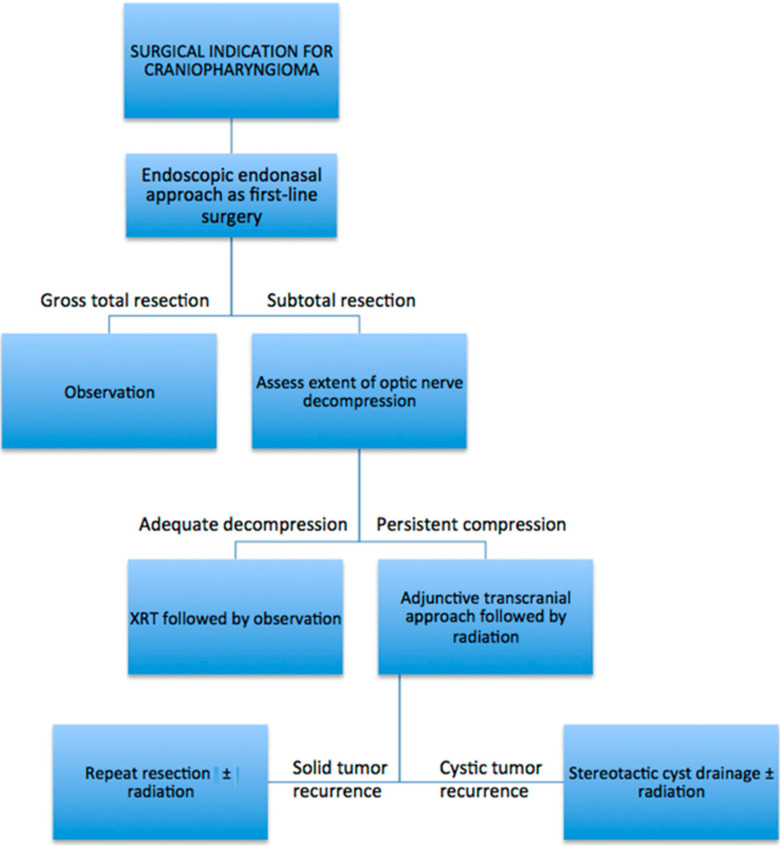
Simplified schematic depicting treatment approach after endoscopic endonasal resection of adult craniopharyngiomas.

At our institution, we routinely utilize the endoscopic endonasal approach as the first surgical intervention for patients with suspected craniopharyngioma diagnosis. The surgical goal is to decompress the optic nerves through a maximally safe resection, given that inadequate separation from the optic nerves may limit adjuvant radiation post-operatively. While GTR is the most desired outcome, it should not be pursued where the tumor cannot be safely removed from the optic nerves, hypothalamus, and pituitary stalk, the latter particularly in patients with normal pre-operative endocrine function. In cases where there is residual tumor, patients are referred routinely for adjuvant radiation therapy, typically with stereotactic radiosurgery. Rarely, the optic nerves are inadequately decompressed after endoscopic surgery, precluding adjuvant radiation. These patients typically have significant lateral extension of their tumors and require a second operation, typically performed during the same admission usually via a supraorbital craniotomy, with the goal of decompressing the optic nerves enough to facilitate post-operative radiation. Other open transcranial approaches are also utilized if the supraorbital approach is not feasible. Patients with papillary craniopharyngiomas harboring BRAF mutations are also evaluated by neuro-oncology for consideration of treatment with oral BRAF inhibitors. Subsequently, after GTR or STR with adjuvant radiation, patients undergo periodic surveillance MRI to monitor for tumor recurrence, as well as routine endocrine evaluations to manage ongoing or delayed endocrine deficits. In patients with tumor recurrence and progression, a repeat resection can be performed for most cases, followed by a combination of re-irradiation and/or chemotherapy. In instances of purely cystic recurrence, stereotactic placement of an Ommaya reservoir into the cyst is preferred, followed by adjuvant radiation during the same hospitalization. While each case of craniopharyngioma is evaluated individually for the most optimal upfront surgical approach or alternative treatment modalities, we have found that the vast majority of operative cases are amenable to an endoscopic endonasal approach. As such, a simplified schematic describing our treatment paradigm for adult craniopharyngiomas undergoing endoscopic resection is shown in Figure 5.

## 6. Conclusions

The management of craniopharyngiomas requires a multi-disciplinary approach involving neurosurgery, otolaryngology, endocrinology, ophthalmology, radiation oncology and neuro-oncology. As highlighted in this review, the introduction and refinement of the endoscopic endonasal approach has revolutionized surgery for craniopharyngiomas, such that the vast majority of these tumors can be resected through this approach. Adjuvant radiation and novel targeted therapies represent viable, effective treatment options for residual tumors after surgery. Likewise, adjunctive open or minimally invasive surgical approaches like a supraorbital craniotomy and/or stereotactic cyst decompression can be utilized to address residual or recurrent tumors. With the success of this multi-modal treatment approach, the goal of surgery remains maximally safe resection, emphasizing preservation of visual and hypothalamic function to maintain quality of life in these patients.

## Figures and Tables

**Table 1:** Key advantages and disadvantages of the endoscopic endonasal approach for craniopharyngiomas.

Advantages	Disadvantages
Minimally invasive leading to faster recovery, shorter hospital stay	Higher rates of CSF leak
Higher rates of gross total resection	Limited reach to purely third ventricular tumors, which may be more amenable unilateral subfrontal or midline transcallosal approaches
Higher rates of improved visual outcomes	Limited reach to tumors with significant lateral extension, which may be more amenable to unilateral pterional/orbitozygomatic/supraorbital approaches
Lower endocrine complication rates	Steep learning curve, may require two surgeons fluent with endoscopic techniques

CSF: (cerebrospinal fluid).
